# Evaluation of sex inequity in lung-cancer-specific survival

**DOI:** 10.2340/1651-226X.2024.27572

**Published:** 2024-05-15

**Authors:** Dan Lærum, Trond-Eirik Strand, Odd Terje Brustugun, Frode Gallefoss, Ragnhild Falk, Michael T. Durheim, Lars Fjellbirkeland

**Affiliations:** a Department of Internal Medicine, Pulmonary Section, Sorlandet Hospital Kristiansand, Kristiansand, Norway; b Department of Patient Safety and Quality, Oslo University Hospital, Oslo, Norway; c Department of Community Medicine, UiT The Arctic University of Norway, Tromsø, Norway; d Section of Oncology, Vestre Viken Hospital Trust, Drammen, Norway; e Faculty of Medicine, Institute of Clinical Medicine, University of Oslo, Oslo, Norway; f Department of Research, Sorlandet Hospital Kristiansand, Kristiansand, Norway; g Medical Faculty, University of Bergen, Bergen, Norway; h Oslo Centre for Biostatistics and Epidemiology, Oslo University Hospital, Oslo, Norway; i Department of Respiratory Medicine, Oslo University Hospital, Oslo, Norway; j Division of Cardivascular and Pulmonary Diseases, Department of Respiratory Medicine, University of Oslo, Oslo, Norway; k Department of Respiratory Medicine, Oslo University Hospital, Oslo, Norway; l Division of Cardiovascular and Pulmonary Diseases, Department of Respiratory Medicine, University of Oslo, Oslo, Norway

**Keywords:** Lung-cancer-specific survival, sex, curative treatment, palliative treatment

## Abstract

**Background:**

Whether sex is an independent prognostic factor in lung cancer survival is the subject of ongoing debate. Both large national registries and single hospital studies have shown conflicting findings. In this study, we explore the impact of sex on lung-cancer-specific survival in an unselected population that is well-characterized with respect to stage and other covariates.

**Material and methods:**

All patients diagnosed with lung cancer at a single hospital serving a whole and defined region in Southern Norway during the 10 years 2007–2016 were included. Follow-up data were available for at least 56 months for all patients. Analyses were adjusted for stage, treatment, performance status, smoking, age, histology, epidermal growth factor receptor/anaplastic lymphoma kinase/immunotherapy treatment and period. Differences in lung-cancer-specific survival by sex were explored using restricted mean survival times (RMST).

**Results:**

Of the 1,261 patients diagnosed with lung cancer, 596 (47%) were females and 665 (53%) males, with mean ages of 68.5 and 69.5 years, respectively. The observed 5-year lung-cancer-specific survival rate was 27.4% (95% CI 23.7, 31.2) in females and 21.4% (95% CI 18.2, 24.8) in males. However, after adjustment for covariates, no significant differences by sex were observed. The 5-year RMST was 0.9 months shorter (95% CI −2.1, 0.31, *p* = 0.26) in males compared to females.

**Interpretation:**

In this cohort, sex was not associated with a difference in lung-cancer-specific survival after adjusting for clinical and biological factors. Imbalance in stage at diagnosis was the main contributor to the observed difference in lung-cancer-specific survival by sex.

## Introduction

In the last decade, the 5-year survival rate for lung cancer patients has nearly doubled in Scandinavia and other countries [1–4]. In Norway, the increase has been from a modest 15% in 2010 to 29.2% in 2021 and applies to both sexes [2]. However, a difference in lung cancer relative survival by sex is evident, with an expected 5-year survival of 25.7% among men versus 32.8% among women in the diagnostic period 2017–2021 [2].

Several epidemiological studies have reported that sex appears to be associated with lung-cancer-specific survival after adjusting for other possible prognostic factors [5–7], leading to suggestions that sex should be considered in treatment decisions [8]. However, the subject remains debatable since other studies have observed no survival difference [9–11]. Several former studies regarding sex-related survival have reported either superior female survival [12–14] or no significant difference [9, 15]. Moreover, most previous studies have been restricted to specific treatments (e.g. surgery [9, 13, 16, 17]), histologies (e.g. adenocarcinoma [7, 18]) or single stages (e.g. stage IV [8]). These former studies may suffer from limitations in reporting survival in only selected groups of patients. There is a lack of studies examining unselected and complete populations across all treatments, histologies and stages, mirroring a real-life population.

To further explore differences in lung-cancer-specific survival by sex, we report results based on data from a single hospital representing a complete, unselected population of lung cancer patients over a 10-year period. Firstly, we aimed to assess whether there is a significant difference in lung-cancer-specific survival between females and males after adjusting for multiple, relevant covariates. Secondly, we determined if sex-related differences in survival were observed in subgroups of patients treated with curative or palliative intent or for patients with adenocarcinoma histology.

## Material and methods

### Study population and inclusion criteria

Virtually all patients with lung cancer in the western part of the administrative region Agder in Norway are diagnosed at Sørlandet Hospital Kristiansand, a community hospital serving a population of 200,000 inhabitants. Fifteen per cent of the population are immigrants with 10% of the total population being non-Caucasian and 5% of Asian descent. Sørlandet Hospital offers free, up-to-date diagnostic investigation and treatment to everyone. There are no other competing options for the initial diagnosis and treatment of lung cancer in the region. During the 10-year period from 2007 to 2016, all patients given a lung cancer diagnosis according to code C34 in the 10th revision of the International Classification of Disease (ICD-10) were registered and added to hospital’s clinical lung cancer database by a single physician and a trained nurse. Patients without tissue-verified diagnosis of lung cancer were included.

### Clinical data

Clinical parameters included sex, age at diagnosis, year of diagnosis (2007–2012 or 2013–2016), smoking status (present, former or never), Eastern Cooperative Oncology Group Performance Status (ECOG PS) (0, 1, 2, 3 or 4), histology (adenocarcinoma, squamous cell carcinoma, small-cell lung carcinoma, no biopsy or other), stage (7th Tumor-Node-Metastasis (TNM) classification was used for all patients, including SCLC) and treatment. Tyrosine-kinase inhibitors (epidermal growth factor receptor (EGFR) or anaplastic lymphoma kinase (ALK) mutation) or immune-oncology therapy were treated as separate binary variables.

Due to a high degree of correlation between stage and treatment, we created a combined variable including combinations of stage and treatment (stage I/surgery, stage I/stereotactic body radiotherapy (SBRT), stage I/palliative treatments (PT) or supportive care only (SCO), stage II/surgery, stage II/SBRT and chemoradiotherapy of curative intent (CRT), stage II/PT or SCO, stage III/surgery, stage III/SBRT and CRT, stage III/PT, stage III/SCO, stage IV/PT or stage IV/SCO) to capture differences in stage and treatment distribution between males and females. In general, patients without biopsy confirmation were also included based on typical morphology on chest CT scanning in case of PT. In patients with curative intent treatment and no biopsy, growth on consecutive CT scans was required in addition to positive FDG-uptake on PET-CT scanning.

The diagnostic year was stratified into 2007–2012 and 2013–2016, due to a local initiative involving diagnostic work-up that was initiated in 2013.

To investigate the second aim, patients were divided into two major treatment groups, curative and palliative intent, defined by the initial treatment received. The former group included patients receiving either surgery, SBRT, CRT or radiation with curative intent (fractionated radiotherapy with cumulative dosage ≥60 Gy). To ensure group uniformity, we included only patients with biopsy-verified lung cancer and ECOG 0–2. The patients treated with palliative intent were divided into two groups. ‘Palliative treatments’ included patients who received palliative chemotherapy and/or radiotherapy. ‘Supportive care only’ included patients who did not receive tumor-specific treatments.

All patients were followed until the end of August 2021. The date of death was available for all patients from the National Population Register of Norway. The underlying cause of death was used to identify deaths related to lung cancer. Only one patient was lost to follow-up.

With the available sample of 1,261 patients, we estimated that we were well within the required sample size of a non-superiority study. This was based on a non-superiority limit of 3 months, i.e. the largest difference in 5-year survival that is clinically acceptable [14]. The standard deviation of the 5-year survival time was set to 9 months. Then, if there is truly no difference between the sexes, 224 patients (both sexes combined) are required to be 80% certain (i.e. power) so that the lower limit of a one-sided 95% confidence interval will be above the non-inferiority limit of 3 months. Thus, many covariates can be adjusted for the analysis that considers the observational design of the data. For the secondary endpoints, the study was also appropriately powered for the subgroups defined by treatment in curative or palliative intent and adenocarcinoma histology.

This study was approved by the Regional Committees for Medical Research Ethics South-East Norway.

### Statistical analysis

Categorical variables are presented as frequencies and percentages and continuous variables as means with standard deviation (SD). Comparison between males and females was performed by students’ *t*-tests for continuous and chi-squared tests for categorical variables.

Lung-cancer-specific survival was defined as the time from diagnosis of lung cancer to date of death due to lung cancer. Patients who died of other causes than lung cancer were censored at the date of death, and patients alive at the end of the study were censored on August 31, 2021 (end of follow-up). Cumulative mortality by cause of death up to 5 years of follow-up was presented separately for males and females.

To explore differences in lung-cancer-specific survival between males and females, we performed restricted mean survival times (RMST) and Cox regression analyses. Because the proportional hazard assumption was unmet, we present RMST as the main analysis. RMST is the mean event-free cause-specific survival time to a prespecified time *t* [19]. It corresponds to the area under the Kaplan–Meier curve up to time *t*, and is a robust measure that is not dependent on proportional hazards. A flexible parametric Royston–Parmar model with adjustment for age, smoking status, period, combined stage and treatment variable, ECOG performance status, histology and EGFR/ALK/immunotherapy treatment was used [20]. The difference in RMST in men compared to women was calculated at 1, 3, and 5 years of follow-up.

To estimate the impact of the difference in stage distribution by sex, we calculated the change in estimate (CIE) of sex as CIE = (O – Z)/O. O (only sex) was the unadjusted estimate of sex using RMST, and Z (sex + stage) was the estimate of sex adjusted for stage. Similar calculations were performed with combined stage and treatment variable instead of only stage.

To investigate the second aim, where we explored sex-related differences between subgroups of patients, stratified analysis was performed with relevant adjustments based on treatment with curative and palliative intent and adenocarcinoma histology. Variable adjustments for the different subgroups are given in Table 3. Thereafter, two sensitivity analyses were performed: (1) A subgroup analysis of patients diagnosed after the quality initiative initiated in 2013 and (2) Cox regression analysis was performed, although the proportional hazard assumption was not met.

Power calculations ensured adequate power in main RMST calculation as well as in included subgroups.

All statistical significance tests were 2-sided, at the 0.05 level. Statistical analyses were performed using Stata, version 16 (StataCorp LLC). The STPM2 function was used for the RMST analysis.

## Results

Of the 1,261 patients diagnosed with lung cancer in the period 2007–2016, 596 (47%) were females and 665 (53%) males (Table 1). Compared to males, females were more frequently diagnosed with stage I disease (25% vs. 17%) and less frequently with stage IV (53% vs. 45%). There was no difference in the use of PET-CT scanning in stages I to III in females (63%) and males (67%). Women received more often treatment with curative intent (40%) compared to men (33%) (*p* = 0.007). Present smokers and never smokers were more common in the female compared to the male populations (54% and 9% vs. 47% and 2%, respectively). In the patients receiving PT, squamous cell carcinoma was more predominant in males, while small cell lung cancer (SCLC) was more common in females. However, significant histological differences were not found in patients receiving curative treatment.

**Table 1:** Characteristics by sex in overall lung cancer population and in patients with curative and palliative intent treatment from 2007 to 2016.

Characteristics	All					Curative					Palliative				
	Women *n* = 596		Men *n* = 665		*P*	Women *n* = 239		Men *n* = 218		*P*	Women *n* = 357		Men *n* = 447		*P*
	*n*	%	*n*	%		*n*	%	*n*	%		*n*	%	*n*	%	
**Age in years, mean (SD)**	68.5	10.9	69.5	10.0	0.09	66.8	8.7	67.1	9.3	0.73	69.7	12.0	70.7	10.1	0.19
**Period**					0.09					0.46					0.21
2007–2012	333	55.9	403	60.6		120	50.2	117	53.7		213	59.7	286	64.0	
2013–2016	263	44.1	262	39.4		119	49.8	101	46.3		144	40.3	161	36.0	
**Smoking**					< 0.001					0.02					< 0.001
Present	323	54.2	312	46.9		128	53.6	100	45.9		195	54.6	212	47.4	
Former	222	37.2	338	50.8		97	40.6	113	51.8		125	35.0	225	50.3	
Never	51	8.6	15	2.3		14	5.9	5	2.3		37	10.4	10	2.2	
**ECOG PS**					0.81					0.57					0.20
0	164	27.5	197	29.6		117	49.0	125	57.3		47	13.2	72	16.1	
1	200	33.6	230	34.6		93	38.9	71	32.6		107	30.0	159	35.6	
2	107	18.0	111	16.7		23	9.6	18	8.3		84	23.5	93	20.8	
3	100	16.7	99	14.9		6	2.5	4	2.8		95	26.6	96	21.5	
4	25	4.2	28	4.2		0	0	0			24	6.7	27	6.0	
**Histology** ^**a**^					0.03					0.36					0.01
Adenoc.	254	42.6	291	43.8		111	46.4	102	46.8		143	40.1	189	42.3	
Squamous cc.	94	15.8	145	21.7		55	23.1	65	29.8		39	10.9	80	17.9	
Small-cell c.	96	16.1	87	13.1		19	7.9	14	6.4		78	21.8	73	16.3	
No-biopsy	99	16.6	91	13.7		24	10.0	18	8.3		75	21.0	73	16.3	
Other	53	8.9	51	7.7		30	12.6	19	8.7		22	6.2	32	7.2	
**TNM stage** ^**b**^					0.002					0.03					0.72
I	151	25.3	115	17.3		137	57.3	98	45.0		14	3.9	17	3.8	
II	49	8.2	63	9.5		41	17.2	55	25.2		8	2.2	8	1.8	
III	128	21.5	134	20.1		60	25.1	61	28.0		68	19.1	73	16.3	
IV	268	45.0	353	53.1		1	0.4	4	1.8		267	74.8	349	78.1	
**Treatment**					0.06					0.69					0.33
Surgery	130	21.8	124	18.6		130	54.4	124	56.9						
SBRT	48	8.1	37	5.6		48	20.1	37	17.0						
CRT	61	10.2	57	8.6		61	25.5	57	26.1						
Palliative treatmentc	223	37.4	294	44.2							223	62.5	294	65.8	
Supportive care only	134	22.5	153	23.0							134	37.5	153	34.2	
**Treatment** EGFR, ALK or immuno					0.27					0.84					0.25
No	574	96.3	632	95.0		230	96.2	209	95.9		344	96.4	423	94.6	
Yes	22	3.7	33	5.0		9	3.8	9	4.1		13	3.6	24	5.4	

Data are shown as *n* (%) except when otherwise specified.

ECOG PS: Eastern Cooperative Oncology Group performance status; SBRT: stereotactic beam radiation therapy; EGFR: epidermal growth factor receptor; ALK: anaplastic lymphoma kinase, SD: standard deviation; adenoc.: adenocarcinoma; squamous cc.: squamous-cell cancer; CRT: chemoradiation therapy.

a Other histology includes NSCLC NOS and carcinoids.

b TNM version 7.

^c^Palliative chemotherapy, radiotherapy or chemoradiotherapy.

**Figure 1:**
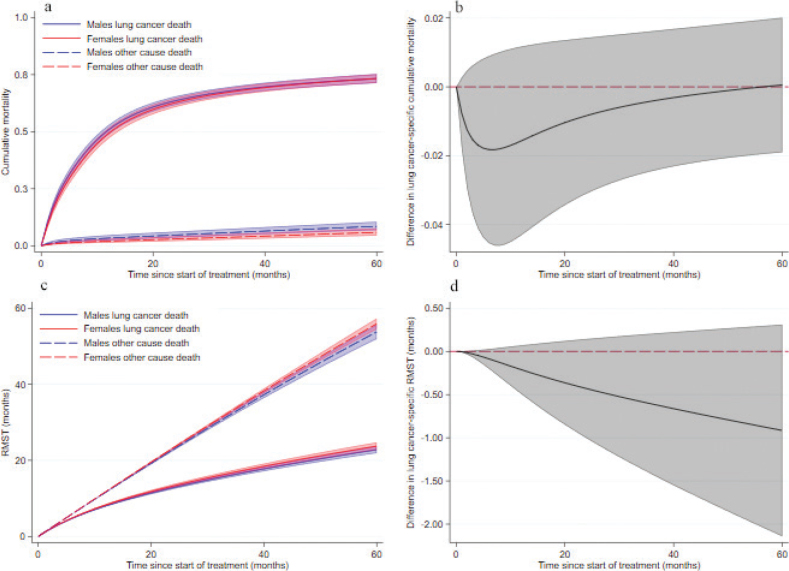
Cumulative mortality and RMST with 95% confidence interval by sex in cohort of lung cancer patients during 5 years of follow-up after start of treatment. (a) Cumulative mortality of lung cancer and other deaths for females and males. (b) Difference in lung-cancer-specific cumulative mortality by sex. (c) RMST of lung cancer death and other death for females and males. (d) Difference in RMST of lung cancer death by sex. RMST: restricted mean survival time.

During follow-up, a total of 916 lung-cancer-specific deaths (498 in males and 418 in females) and 105 deaths due to other causes than lung cancer (58 in males and 47 in females) occurred. Unadjusted 1-year lung cancer-specific survival was 47.7% (95% confidence interval [CI] 43.8, 51.5) in males and 54.5% (95% CI 50.4, 58.4) in females. The corresponding 5-year survival was 21.4% (95% CI 18.2, 24.8) and 27.4% (95% CI 23.7, 31.2), respectively. The cause-specific cumulative mortality by sex is presented in Figure 1a. No difference between sexes was observed in lung-cancer-specific cumulative mortality (Figure 1b).

Unadjusted lung-cancer-specific RMST was significantly shorter in males compared to females at 5 years (−3.6 months, 95% CI −6.2, −1.0) (Table 2).

**Table 2:** Lung-cancer-specific RMST in males compared to females.

Adjustments	Follow-up	Difference in RMST (months)	95% Confidence Interval	*P*
Unadjusted	1 year	−0.52	−0.89, −0.15	0.03
	3 years	−2.1	−3.6, −0.61	0.01
	5 years	−3.6	−6.2, −1.0	0.006
Adjusted^a^	1 year	−0.21	−0.50, 0.07	0.19
	3 years	−0.61	−1.4, 0.21	0.35
	5 years	−0.92	−2.1, 0.31	0.26

Difference in lung-cancer-specific RMST in males and females at 1, 3 and 5 years. Negative numbers indicating shorter survival in males. RMST: restricted mean survival time.

a Adjusted for combined stage and treatment group, Eastern Cooperative Oncology Group Performance Status (ECOG PS), smoking, age, histology, EGFR/ALK/immunotherapy treatment and period.

There was no difference in lung-cancer-specific RMST by sex after adjusting for covariates (Table 2, Figures 1c and 1d). At 1, 3 and 5 years, males had 0.2 months (95% CI −0.5, 0.1), 0.6 months (95% CI −1.4, 0.2) and 0.9 months (95% CI −2.1, 0.3) shorter lifetime expectancy than females, although not statistically significant.

The estimated impact of adding information about stage to the unadjusted model, including only sex, showed a change from −3.6 to −0.1 months in the 5-year RMST, corresponding to a CIE of 98%. Similar calculations for the combined stage and treatment variable showed a CIE of 83%.

Stratified analysis by treatment intent showed no statistical differences by sex. In a well-defined population of patients treated with curative intent (surgery, SBRT or CRT) with tissue-verified lung cancer and ECOG 0–2, no difference in survival was found (−0.1 months, 95% CI −3.4, 3.2) at 5 years (Table 3). Similarly, in patients receiving PT or SCO, no difference was found at 2 years (−0.9 months, 95% CI −1.9, 0.2).

**Table 3:** Lung-cancer-specific RMST in males compared to females treated with curative or palliative intent and for different histologies.

Stratification	*N*	Follow-up	Difference in RMST (months)	95% confidence interval
**Treatment intent**				
Curative treatment^a,c^	409	5 years	-0.07	-3.4, 3.2
Palliative treatment^b,c^	804	2 years	-0.88	-1.9, 0.16
**Histology**				
Adenocarcinoma^d^	545	5 years	-1.0	-2.9, 0.88

a Surgery, stereotactic beam radiation therapy or chemoradiation with curative intent, ECOG 0–2 and histology confirmed lung cancer. RMST: restricted mean survival time.

b Palliative chemotherapy, radiotherapy, chemoradiotherapy or supportive care only.

c Adjusted for combined stage and treatment group, Eastern Cooperative Oncology Group Performance Status (ECOG PS), smoking, age, histology, EGFR/ALK/immunotherapy treatment and period.

d Adjusted for the same as ^c^ except histology.

Stratification according to the histological subgroup adenocarcinoma also revealed no significant differences by sex.

In sensitivity analyses, results of univariate Cox regression analysis suggested higher mortality among males compared to females (hazard ratio [HR] 1.2, 95% CI 1.1, 1.4) (Table 4). However, after adjusting for covariates, no significant difference was observed (HR 1.1, 95% CI, 0.96, 1.25).

## Discussion

In this study, with complete data from an unselected and defined population of lung cancer patients over a time span of 10 years, we have studied the impact of sex as an independent factor affecting survival. Although unadjusted lung-cancer-specific survival at 1-year and 5-year survival was superior in women compared to men, this difference disappeared after adjustments. It seemed to be explained by selected clinical variables, in particular stage at presentation. A recent Australian study [21], supports our findings by showing a large reduction in male excess dying after adjusting for several prognostic factors.

In our study, the unequal stage distribution with more early-stage cancer in females and late-stage cancer in males was the major factor accounting for differences in lung-cancer-specific survival. Previous studies have suggested that sex inequity could be explained by differences in health-seeking behavior [22, 23]. The great majority of stage I tumors are asymptomatic and discovered due to imaging procedures performed for other reasons [24]. Other studies have indicated that females are generally more prone than men to utilize medical care [25, 26]. However, there are to our knowledge no studies linking sex-related differences in health awareness or healthcare utilization specifically to stage at presentation in lung cancer.

Several previous single hospital studies [8, 10, 27] or larger population-based cohort studies [5, 7, 28] conclude that there is an independent survival benefit in females.

While single hospital studies could be limited by selection bias, larger registry studies may often include less detailed clinical data and thus have limited ability to adjust for important factors affecting survival in males and females. This study combines the detail level of single-hospital series with the benefit of an unselected cohort as population-based studies could offer. In a large-scale national study (>40,000 lung cancer cases) based on data from the Cancer Registry of Norway, Sagerup et al. [5] found a higher relative risk of death in males (HR 1.14). This study including patients from 1988 to 2007 had an uneven distribution of sexes with approximately 1/3 female and 2/3 male, corresponding to different exposure to cigarette smoking. Diagnostic and staging modalities were scarcer and unevenly distributed in Norway in that time period. Furthermore, the stage distribution used by Sagerup was different and more unrefined, not using the TNM-system, but recorded at the cancer registry as either ‘localized’, ‘regional’ or ‘distant’, based on the best available clinical information they received. Neither performance status nor treatment information was available, both of which are possible factors affecting survival in lung cancer. A similar large-scale study based on data from the neighboring Swedish Lung Cancer Registry (2002–2016) also found poorer prognosis in males, particularly those with adenocarcinoma [7]. A wide range of prognostic factors were adjusted for. Still, only patients with verified adenocarcinoma and squamous cell cancer were included, excluding other histologies and non-biopsied patients with clinical lung cancer diagnosis. Patients were staged according to different TNM versions, TNM 6th in the first part of the cohort and 7th edition in the latter, and treatment was not included in the final analysis. Both the Norwegian and the Swedish data sets are large national registry-based studies that have some limitations with respect to prognostic factors that might affect adjusted analyses.

We present a complete cohort, including every patient diagnosed with lung cancer in our region. Our cohort is a real-life population of lung cancer patients where as much prognostic information as possible is retained to minimize confounding in interpreting sex and its influence on survival [29]. Heterogeneity in histology, treatment and stage was properly adjusted for in the calculations. The population also includes similar numbers of males (53%) and females (47%), from a country where the percentage of daily smokers has been similar in males and females since the mid-1990s. Furthermore, in Norway, a universal healthcare insurance system financed by taxation ensures coverage of all citizens. This allows equal access to health services, including diagnostics and lung cancer treatment, independent of social status, age or address. Norway is ranked 3rd in the world in gender equality, limiting differences by sex that are not detected by clinical parameters [30].

To further reflect real-world conditions, unbiopsied patients with a clinical lung cancer diagnosis were included. This latter group of the cohort, constituting 15% of the total, consists of patients of older age, complex comorbidity and poor performance status. It also includes 40 patients of older age and/or poor lung function who, based on morphological diagnosis of lung cancer, received curative intent treatment with SBRT for their stage I disease. Virtually all patients in Norway are registered in the Cancer Registry of Norway, including patients without tissue biopsy-proven diagnosis. This latter group of patients were included in the analysis as we intended to examine differences in sex-related survival in a complete lung cancer population. One-fifth of our cohort had poor performance status, ECOG 3–4, a population that is often lacking in other studies. Performance status is an independent predictor of lung-cancer-specific survival and should be included in order to avoid misinterpretation of apparent differences in sex-related survival [31]. It has been shown that ECOG may predict outcome of lung cancer better than comorbidity [32].

Both stage and treatment modalities are important factors influencing survival in lung cancer. In multivariate analysis, multicollinearity arises between these two factors, forcing one of the two to be removed from the analysis. To avoid losing prognostic information, we created combined stage and treatment groups. This distinguishes our study from other similar studies and might be an explanatory factor for our finding of similar lung-cancer-related mortality in males and females.

In Norway, the incidence of lung cancer in females and males is now equal, and at the same time, smoking prevalence over the last decades has changed. In 1975 there were 20 percentage points more daily male smokers compared to the female population aged 16–74 years (50% vs. 30%) [33]. The gap diminished gradually and was eliminated in the mid-1990s, with 31% daily smokers in both populations, followed by a gradual reduction to 9% for both sexes in 2020. In our cohort, 54% of women and 47% of men were active smokers at diagnosis. Although this is a small numerical difference, it reverses the historical distribution of male excess smoking. In 2010 in Norway, less than 20% of daily and occasional smokers used non-cigarette types of tobacco, including mainly cigars and less pipe tobacco. Duration and intensity of cigarette smoking show a dose-dependent risk in the development of lung cancer [34].

Analysis of survival in subgroups (Table 3) also did not reveal sex-related differences. We were not able to find other reports examining sex-specific survival in the group of curative intent treatment taking into account the three separate treatments: surgery, SBRT or curative intent CRT. In our cohort, curative intent treatment was initiated among 40% of females, which was more frequent than in males (33%). This is as expected from the observed stage differences. However, there was no difference in lung-cancer-specific survival between males and females treated with curative intent. Other studies report adenocarcinoma being more common in females [13, 14]. Superior survival of adenocarcinoma compared to other histologies has been suggested as one explanatory factor of survival benefit in females [12, 13]. We observed an equal incidence of adenocarcinoma in males and females, and after adjusting for relevant covariates, there was no survival benefit in adenocarcinoma compared to other histologies.

The results were robust to the statistical method used. Both RMST and Cox-regression analysis did not reveal any statistically significant difference in female and male lung-cancer-specific survival after adjusting for relevant clinical factors. Just as important, our observed difference of 0.9 months between females and males at 5 years, a 1% difference, is far from what would be considered a clinically meaningful difference [35].

Among the limitations of this study is the retrospective nature of the first period of the cohort, which may introduce information bias. We do not expect this to have distorted interpretations of sex-related differences. Our data were not detailed enough to allow differentiating according to type and total consumption of tobacco. There was also incomplete data regarding workplace exposure. Including these parameters would have added additional background information. If the effects of these data would have been strong enough to influence our survival findings is thus an open question. The study population comes from a single hospital, which may limit the validity of our findings. Preferably, to confirm our findings, the study should be repeated in another population. Furthermore, the population in our region is mainly Caucasian (>90%), whereas 5% are of Asian origin. Due to differences in genetic susceptibility, exposure, molecular profiles, and clinical outcomes in East-Asian versus Caucasian populations, the study has lower external validity with respect to Asian populations [36]. Finally, analyses were not adjusted for comorbidity and social status as these data were lacking.

## Conclusion

In this complete cohort of 1,261 lung cancer patients, the superior unadjusted survival seen in females does not persist after adjusting for biological and clinical factors. We conclude that the apparent survival advantage in females is not due to the difference in sex-related biological behavior of lung cancer, but can be explained by differences in other clinical parameters where stage at presentation remains most important.

## Declarations

### Ethics approval and consent to participate

This study was approved by the regional Committees for Medical Research Ethics South-East Norway (reference number 405718-2022). Informed consent was deemed unnecessary by the regional Committees for Medical Research Ethics according to Norwegian legislation by authority of the Norwegian Health Personnel Act § 29 and the Health Research act § 10.

All methods were carried out in accordance with relevant guidelines and regulations in the declaration – Ethics approval and consent to participate section.

## Consent for publication

Not applicable.

## Data availability statement

The datasets generated and/or analyzed during this study are not publicly available due to national regulations. Access to the dataset was restricted to the authors of the article, and sharing of the data would require new applications to the regional Committees for Medical Research Ethics South-East Norway. Further details on the data are available from the corresponding author on reasonable request.

## Competing interests

The authors report there are no competing interests to declare.

## Author contributions

CRediT author statement.

D.L.: Conceptualization, Methodology, Formal analysis, Writing – original draft, Visualization. O.T.B.: Writing – review and editing, Conceptualization F.G.: Writing – review and editing and Conceptualization. R.F.: Writing – review and editing, Validation. T.-E.S.: Writing – review and editing. M.T.D.: Writing – review and editing. L.F.: Writing – original draft, review and editing, Conceptualization, Supervision.
